# Efficacy and cost-utility of the eHealth application ‘Oncokompas’, supporting patients with incurable cancer in finding optimal palliative care, tailored to their quality of life and personal preferences: a study protocol of a randomized controlled trial

**DOI:** 10.1186/s12904-019-0468-8

**Published:** 2019-10-23

**Authors:** Anouk S. Schuit, Karen Holtmaat, Nienke Hooghiemstra, Femke Jansen, Birgit I. Lissenberg-Witte, Veerle M. H. Coupé, Myra E. van Linde, Annemarie Becker-Commissaris, Jaap C. Reijneveld, Josée M. Zijlstra, Dirkje W. Sommeijer, Simone E. J. Eerenstein, Irma M. Verdonck-de Leeuw

**Affiliations:** 1 Department of Clinical, Neuro and Developmental Psychology, Faculty of Behavioral and Movement Sciences, Amsterdam Public Health Research Institute, Vrije Universiteit Amsterdam, van der Boechorststraat 7, 1081 BT Amsterdam, The Netherlands; 2 Cancer Center Amsterdam (CCA), Amsterdam UMC, Vrije Universiteit Amsterdam, Amsterdam, The Netherlands; 3 Amsterdam UMC, Vrije Universiteit Amsterdam, Otolaryngology – Head and Neck Surgery, Cancer Center Amsterdam, De Boelelaan, 1117 Amsterdam, The Netherlands; 4 Department of Epidemiology and Biostatistics, Amsterdam UMC, Vrije Universiteit Amsterdam, De Boelelaan, 1117 Amsterdam, The Netherlands; 5 Department of Medical Oncology, Cancer Center Amsterdam, Amsterdam UMC, Vrije Universiteit Amsterdam, De Boelelaan, 1117 Amsterdam, The Netherlands; 6 Department of Pulmonary Diseases, Cancer Center Amsterdam, Amsterdam UMC, Vrije Universiteit Amsterdam, De Boelelaan, 1117 Amsterdam, The Netherlands; 7 Department of Neurology, Cancer Center Amsterdam, Amsterdam UMC, Vrije Universiteit Amsterdam, De Boelelaan, 1117 Amsterdam, The Netherlands; 8 Department of Hematology, Cancer Center Amsterdam, Amsterdam UMC, Vrije Universiteit Amsterdam, De Boelelaan, 1117 Amsterdam, The Netherlands; 9 Department of Internal Medicine, Cancer Center Amsterdam, Amsterdam UMC, University of Amsterdam, Meibergdreef 9, Amsterdam, The Netherlands; 10 Department of Internal Medicine, Flevo Hospital, Hospitaalweg 1, Almere, The Netherlands

**Keywords:** Incurable cancer, Palliative care, Supportive care, eHealth, Self-management, Patient activation

## Abstract

**Background:**

Patients with incurable cancer have to deal with a wide range of symptoms due to their disease and treatment, influencing their quality of life. Nowadays, patients are expected to adopt an active role in managing their own health and healthcare. Oncokompas is an eHealth self-management application developed to support patients in finding optimal palliative care, tailored to their quality of life and personal preferences. A randomized controlled trial will be carried out to determine the efficacy and cost-utility of Oncokompas compared to care as usual.

**Methods:**

136 adult patients with incurable lung, breast, colorectal and head and neck cancer, lymphoma and glioma, will be included. Eligible patients have no curative treatment options and a prognosis of at least three months. Patients will be randomly assigned to the intervention group or the control group. The intervention group directly has access to Oncokompas alongside care as usual, while the waiting list control group receives care as usual and will have access to Oncokompas after three months. The primary outcome measure is patient activation, which can be described as a patient’s knowledge, skills and confidence to manage his or her own health and healthcare. Secondary outcome measures comprise self-efficacy, health-related quality of life, and costs. Measures will be assessed at baseline, two weeks after randomization, and three months after the baseline measurement.

**Discussion:**

This study will result in knowledge on the efficacy and cost-utility of Oncokompas among patients with incurable cancer. Also, more knowledge will be generated into the need for and costs of palliative care from a societal and healthcare perspective.

**Trial registration:**

Netherlands Trial Register identifier: NTR 7494. Registered on 24 September 2018.

## Background

Quality of life is an important aspect of healthcare for patients with incurable cancer. These patients have to deal with physical symptoms due to their disease and treatment, and often suffer from psychological, social and existential concerns, negatively affecting their quality of life [1–3]. Palliative care (or supportive care) for patients with incurable cancer focuses on reducing symptoms, improving quality of life and supporting patients and their families [4]. It not only concerns the management of physical symptoms related to the disease and its treatment. It also involves the provision of services to meet emotional, social, psychological, spiritual, informational, and practical needs [5–7]. Although there is evidence that early palliative care improves patients’ quality of life [8], palliative care services are often discussed at a late stage of the advanced cancer trajectory and many patients have unmet needs [9, 10].

Nowadays, patients are expected to adopt an active role in the management of their own well-being and healthcare [9, 10]. Self-management is defined as “those tasks that individuals undertake to deal with the medical, role, and emotional management of their health condition(s)” [11]. Research has shown that interventions supporting self-management can improve quality of life of patients with chronic disease and can be cost-effective [13–15]. They can also be beneficial for patients in terms of self-efficacy and patient activation [11, 16]. Evidence suggests that cancer patients with high self-efficacy are less likely to have negative psychological outcomes [17].

Patient activation can be described as a patient’s knowledge, skills and confidence to manage his or her own health and healthcare [18]. Research indicated that changes in activation are followed by changes in self-management behaviors [16] and that more activated patients are less likely to have unmet needs [19]. A study among patients with diabetes reported the positive relation between patient activation and self-reported health status across several studies [20]. Furthermore, a higher level of patient activation is associated with lower total costs from a healthcare and societal perspective [21]. Patient outcomes may be influenced by patients’ confidence to manage their disease and thereby lead to lower healthcare costs [22].

Self-management can be stimulated through the use of eHealth. A systematic review showed evidence for positive effects of eHealth on cancer patients’ knowledge levels and information competence, and possibly also on health status and quality of life [12]. Furthermore, eHealth has the potential to be cost-saving [23]. To the authors’ knowledge there is no clear evidence on the efficacy of tailored eHealth interventions supporting self-management in palliative care.

To support cancer patients in managing their well-being by informing them where they can find advice and guidance, the eHealth self-management application Oncokompas has been developed. This application helps patients to monitor their quality of life, using Patient-Reported Outcome Measures (PROMs), followed by automatically generated feedback and advice on palliative care services, tailored to their health status and personal preferences. The aim of the current study is to determine the efficacy and cost-utility of Oncokompas as a self-management instrument on patient activation, general self-efficacy, and quality of life among patients with incurable cancer (who are not yet in the terminal phase of their illness) compared to care as usual.

## Methods/design

### Study design

A prospective monocenter randomized controlled trial (RCT) with two parallel groups will be conducted among patients with incurable cancer to determine the efficacy and cost-utility of Oncokompas.

Patients will be randomly assigned to the intervention group or the waiting list control group. Patients in the intervention group will get direct access to Oncokompas alongside care as usual, while patients in the control group will receive care as usual and will be placed on a waiting list. This means that they will be given access to Oncokompas three months after the baseline measurement (i.e. after completion of the last questionnaire (t2)).

**Fig. 1:**
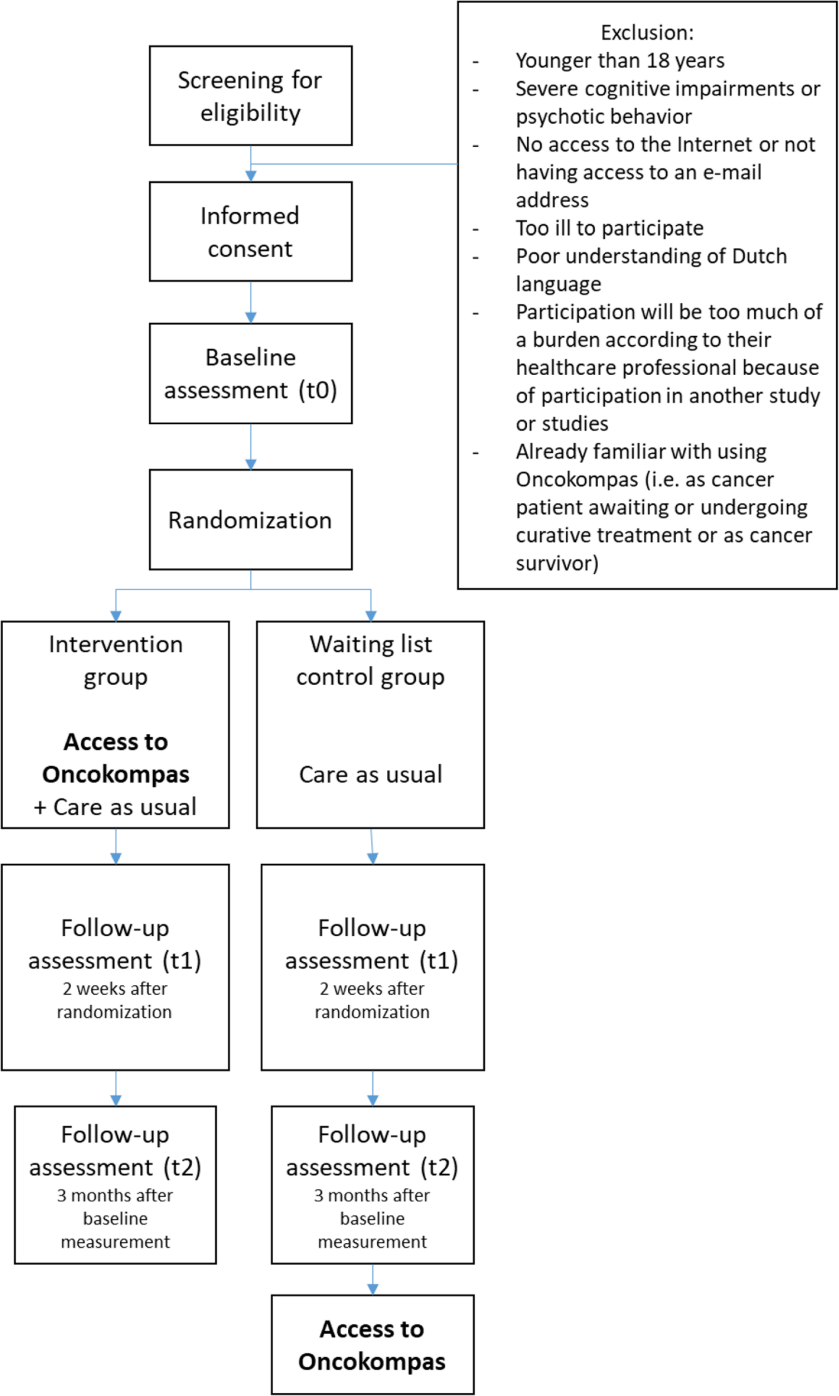
Flow diagram of the RCT

**Fig. 2:**
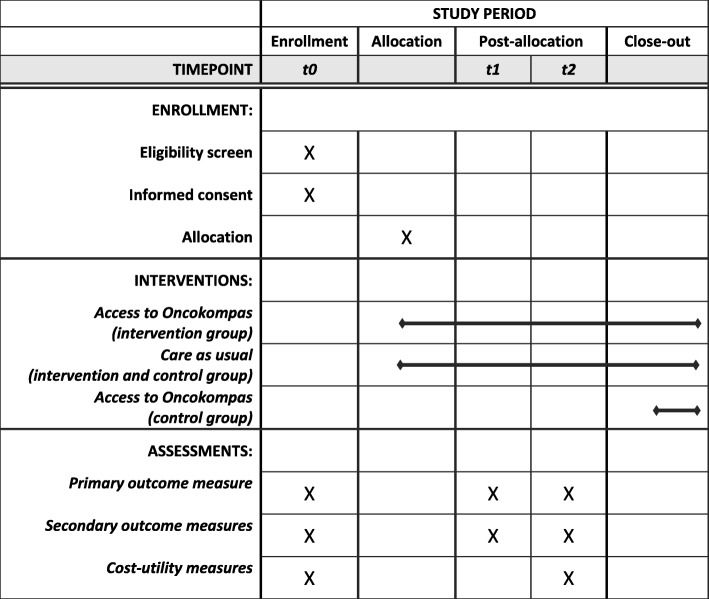
The schedule of enrollment, intervention and assessments of the RCT (according to SPIRIT)

This study has been approved by the VUmc Medical Ethical Committee (registration number 2018.224). All respondents are informed that participation is voluntary. Respondents will provide written informed consent before inclusion. The flow diagram of the RCT is shown in Fig. 1. Figure 2 shows the schedule of enrollment, intervention and assessments (according to the Standard Protocol Items: Recommendations for Intervention Trials (SPIRIT)) (see Additional file 1).

### Study population

#### Inclusion and exclusion criteria

This study will include adult patients (18 years or older) with incurable cancer (i.e. not having curative treatment options) who have a life expectancy of at least three months. Patients are included when they are diagnosed with lung cancer, breast cancer, colorectal cancer, head and neck cancer, lymphoma, or glioma. Furthermore, patients must be aware of the incurability of their cancer.

Patients are excluded when they have severe cognitive impairments or psychotic behavior (delusions and hallucinations), a poor understanding of the Dutch language (and thereby are not able to complete a Dutch questionnaire), or when they are too ill to participate. Patients are also excluded when they do not have access to the Internet or do not have access to an e-mail address, when their healthcare professional thinks that participation will be too much of a burden because a patient is already participating in other studies, or when they already used Oncokompas before (i.e. as cancer patient awaiting or undergoing curative treatment, or as cancer survivor).

### Study procedures

In various hospitals in the Netherlands, patients will be informed about this study by their healthcare professional. Patients eligible to participate will be approached by their medical specialist, (research)nurse or nurse specialist when they visit the outpatient clinic. Apart from informing and referring patients to the research team, no actions regarding the study will take place in the hospitals (therefore this study is marked as a monocenter study).

The coordinating researcher will further inform interested patients by phone or through direct face-to-face contact at the outpatient clinic. Patients also receive a letter with information about the study and Oncokompas. When patients want to participate, they sign the informed consent form. After the researcher has received the informed consent form, patients will receive a link to the online baseline questionnaire by e-mail. Patients who completed the baseline questionnaire (t0) will be randomized into the intervention group or the control group. Patients randomized in the intervention group will receive an invitation e-mail for Oncokompas through which they can activate their personal account. Patients randomized in the control group will receive an e-mail to activate their Oncokompas account after completion of the last questionnaire (t2). The other questionnaires will be sent two weeks after randomization (t1) and three months after the baseline measurement (t2).

#### Randomization

After completion of the baseline questionnaire, patients are randomly assigned to the intervention group or the control group, using block randomization. Blocks will have a length of four up to eight. Randomization takes place in a 1:1 ratio. The randomization scheme is created by a researcher not involved in the study, which also carries out the allocation of participants, using random allocation software (i.e. Sealed Envelope). Subsequently, this researcher notifies the coordinating researcher of the study about the outcome of the allocation after randomizing a participant. Blinding of the coordinating researcher is not possible since this researcher will send out the invitations for Oncokompas to patients and has to support patients during the study, for example when they have questions regarding technical issues.

### Intervention

Oncokompas is an eHealth self-management application that supports patients in finding and obtaining optimal palliative care, tailored to their health status, personal characteristics and preferences.

Oncokompas comprises three components: 1) Measure, 2) Learn, and 3) Act.

After the log-in procedure is completed, patients enter the first component of Oncokompas, ‘Measure’. This component starts with the completion of a general questionnaire. Based on the patient’s answers, Oncokompas selects the topics appropriate for this patient (e.g. when someone has no children, there will be no children-related topics). Subsequently, patients can select which topics they want to monitor within Oncokompas. Table 1 gives an overview of all the topics covered in Oncokompas. Patients complete Patient-Reported Outcome Measures (PROMs) on the topics they have chosen. Patients can complete PROMs targeting different domains of quality of life; physical, psychological and social functioning, and existential issues. PROMs were selected based on Dutch practical guidelines and literature searches, in collaboration with healthcare professionals and patients. The answers given to the PROMs are processed real-time and algorithms are used to link them to feedback in the ‘Learn’ component. All algorithms are based on available cut-off scores, Dutch practical guidelines and/or consensus by teams of experts.

**Table 1:** Overview of all topics covered in Oncokompas for patients with incurable cancer

Domain	Topics
Physical	Body weight
	Daily functioning
	Diarrhea
	Dysphagia
	Dyspnea
	Fatigue
	Information about treatment options
	Appetite loss
	Lymphedema
	Mouth problems
	Nausea and vomiting
	Obstipation
	Pain
	Sexuality
	Skin problems
	Sleep problems
	Other side effects of medical treatment
Psychological	Cancer related anxiety (including fear of suffering and fear of dying)
	Coping with emotions
	Depression
	Tenseness
Social	Being single and cancer
	Choices concerning the end-of-life
	Loneliness
	Meaningful daily activities
	Patient-physician communication
	Social life
	Relationship with partner
	Relationship with (adult) children
	Work issues
Existential	Meaning of life
	Saying farewell

In the ‘Learn’ component, patients get an overview of their overall well-being on topic level, using a three-color system. A green score means that the patient is doing well on a topic, an orange score means that a topic could use attention and support, and a red score means that a topic needs attention and support. Patients get personalized feedback on their outcomes, tailored to their health status, personal characteristics and preferences. In addition, Oncokompas provides information on evidence-based interrelated symptoms (e.g. depression and sleeping problems). The feedback in the ‘Learn’ component concludes with comprehensive self-care advice, such as tips and tools, tailored to the individual patient.

In the ‘Act’ component, patients are provided with personalized palliative care options, based on their health status, preferences (e.g. preferences for individual therapy versus group therapy) and their neighborhood (e.g. Oncokompas shows the palliative care options the closest to the patient, based on a patient’s ZIP code). When patients have an orange score on a topic, the feedback includes suggestions for self-help interventions. When they have a red score on a topic, the feedback always includes the advice to contact their medical specialist, general practitioner, or a specialized healthcare professional (e.g. a physiotherapist or psychologist) [24].

Initially, Oncokompas was developed targeting cancer survivors [24–26]. From 2016 till 2018 Oncokompas has been extended to make the content of the application suitable for patients with incurable cancer, who are not yet in the terminal phase of their illness. The content of Oncokompas is developed in cooperation with patients, healthcare professionals and representatives of allied health services, using a stepwise, iterative, and participatory approach. This method actively involves users and other stakeholders in the design process [27].

### Care as usual

In this study, care as usual is defined as the care provided by the oncological team or by other healthcare professionals. This includes all medical and palliative care that patients receive, regardless of their participation in this study.

### Outcome assessment

The primary outcome measure to assess the efficacy of Oncokompas is patient activation. Secondary outcome measures are general self-efficacy and health-related quality of life. Also cost-utility outcomes will be evaluated. Outcome measures will be collected through online questionnaires at baseline (t0), two weeks after randomization (t1), and three months after the baseline measurement (t2).

An overview of the primary and secondary outcome measures is shown in Table 2.

**Table 2:** Measurement overview

Aim	Outcome measures	Time point		
	*Instrument*	Baseline (t0)	Two weeks after randomization (t1)	Three months after baseline measurement (t2)
Efficacy				
*Primary outcome measure*	Patient Activation	X	X	X
	*Patient Activation Measure (PAM)*			
*Secondary outcome measures*	Self-efficacy	X	X	X
	*General Self-Efficacy Scale (GSE)*			
	Health-related quality of life	X	X	X
	*EORTC QLQ-C15 PAL*			
Cost-utility				
	Quality-adjusted life years	X		X
	*EuroQol 5 Dimensions (EQ-5D)*			
	Medical costs	X		X
	*iMTA Medical Consumption* * Questionnaire (iMCQ)*			
	Productivity costs	X		X
	*iMTA Productivity Cost Questionnaire (iPCQ)*			

### Primary outcome measure

#### Patient activation

Patient activation is measured with the Patient Activation Measure (PAM) [16, 26–29]. This questionnaire measures a patient’s self-reported knowledge, skills and confidence for self-management of his or her health or chronic condition [18]. The PAM consists of 13 items with a 4-point Likert scale on which patients can report their level of agreement (i.e. strongly disagree, disagree, agree, and strongly agree) or indicate that the item is not applicable. There are four levels of patient activation, ranging from the patients who hardly feel in charge of their own health (level one) to the patients who think they are well capable to manage their own health and healthcare (level four).

The total PAM score is computed by calculating the mean score of all the applicable items and transforming the mean score to a standardized activation score ranging from 0 to 100 [30]. Non-applicable items are not taken into account to calculate the mean score. Higher total PAM scores indicate a higher level of patient activation. The psychometric properties of the PAM 13-Dutch are generally good; the level of internal consistency is good (Chronbach’s alpha = 0.88) and item-rest correlations are moderate to strong [30].

### Secondary outcome measures

#### General self-efficacy

The General Self-Efficacy Scale (GSE) is a unidimensional questionnaire designed to assess how a person deals with difficult situations in his or her life. The GSE consists of 10 items with 4-point Likert scales ranging from 1 up to 4 (i.e. not at all true, hardly true, moderately true, and exactly true). The total score is calculated by adding up the scores on the 10 items, ranging from 10 to 40. A higher total GSE score indicates higher self-efficacy [31]. The psychometric properties of the GSE have been examined among participants from 25 countries; Cronbach’s alphas ranged from 0.76 to 0.90, with the majority in the high 0.80s [32].

#### Quality of life

Quality of life is measured by the European Organization for Research and Treatment of Cancer Quality of Life Questionnaire for cancer patients in palliative care (EORTC QLQ-C15-PAL). The EORTC QLQ-C15-PAL is an abbreviated 15-item version of the EORTC QLQ-C30 questionnaire. The EORTC QLQ-C15-PAL questionnaire is specifically designed for patients with advanced, incurable and symptomatic cancer with a median life expectancy of a few months [33].

The EORTC QLQ-C15-PAL comprises a global quality of life scale, two functional scales (physical and emotional functioning), two symptom scales (fatigue and pain), and four single items (dyspnea, insomnia, appetite loss, and constipation) [33]. All scales range in score from 0 to 100. A high score on the global quality of life scale represents a high quality of life and a high score on a functional scale represents a high or healthy level of functioning. A high score on a symptom scale indicates a high level of symptoms [34].

##### Cost-evaluation

A cost-utility analysis will be conducted comparing the difference in total three-month costs between the two study arms to the difference in Quality-Adjusted Life Years (QALYs) based on the EuroQol 5 Dimensions (EQ-5D).

#### EuroQol 5 dimensions

The EuroQol 5 Dimensions (EQ-5D-5L) asks respondents to describe their health state on five dimensions of quality of life (i.e. mobility, self-care, usual activities, pain/discomfort, and anxiety/depression). All those dimensions split into five levels. As a result, there are 3125 possibilities for one’s health status. The profile of answers that results after completing the questionnaire can be transformed to a given answer by the general public: the EQ-5D index using the Dutch index tariff. The EQ-5D also includes a visual analogue scale from 0 (worst health state) to 100 (best health state) on which respondents can represent their own health state. The EQ-5D is a validated instrument to measure health-related quality of life [35].

#### Medical consumption questionnaire and productivity cost questionnaire

An adapted version of the medical consumption questionnaire (iMCQ) and productivity cost questionnaire (iPCQ) will be used to measure the costs of healthcare (i.e. healthcare use and medication use), the costs for patients and their families (e.g. travelling costs and help received from family or friends), and costs within other sectors (e.g. productivity losses from paid work) in the previous three months. Both questionnaires are developed by the Institute for Medical Technology Assessment of the Erasmus University Rotterdam (iMTA), the Netherlands [36, 37].

#### Sociodemographic and medical data

Sociodemographic and clinical characteristics (i.e. age, gender, education level, and work situation) will be assessed at baseline (t0) using a study-specific questionnaire. Other characteristics (i.e. cancer type, treatment modality, and time since treatment) will be collected from the hospital information system, using a study-specific case report form.

### Sample size

To demonstrate the presence of an effect on the PAM between t0 and t2 of at least 0.5 standard deviations as statistically significant in a one-tailed test at alpha = 0.05 and a power of (1 - beta) = 0.80, at least 51 participants in each condition will be required at three months follow-up. Anticipating a dropout rate of 25% between t0 and t2 (based on earlier research in this population [38]), 68 participants per condition arm need to be included at baseline (t0). In total, 136 cancer patients will be recruited for this study.

### Statistical analyses

All analyses will be conducted according to the intention-to-treat principle. Descriptive statistics will be generated to describe all sociodemographic and clinical characteristics, and outcome measures. To analyze whether randomization resulted in a balanced distribution of patient characteristics across the study arms, chi-square tests and independent samples t-tests will be used. When data is not normally distributed, Mann-Whitney U tests will be performed. In addition, independent samples t-tests will be used to test whether there are differences in outcome measures across study arms at baseline.

Linear Mixed Models (LMM) will be used to determine the efficacy of Oncokompas (e.g. changes in patient activation in the intervention group and the control group between t0, t1, and t2) by comparing longitudinal changes between both groups with fixed effects for study arm, time, and their two-way interaction, as well as a random intercept for subjects, and, if necessary, for referring hospitals. In case of baseline differences between study arms in sociodemographic and clinical characteristics, or outcome measures, the LMM analyses will be corrected for these differences. LMM will also be used to determine whether age, gender, socio-economic status (e.g. education level and work situation), cancer type, treatment modality, time since treatment, and baseline quality of life moderate the efficacy of Oncokompas. Fixed effects will be used for study arm, time, the potential moderator, and all two-way and three-way interaction effects, as well as a random intercept for subjects, and, if necessary, for referring hospitals.

Post-hoc analyses will be applied when significant results are found in the efficacy and moderation analyses mentioned above. Independent samples t-tests with Bonferroni correction will be used to measure the differences between the intervention group and the control group at follow-up measurements. To measure the effect sizes (ES) of the intervention, the (between group) Cohen’s d will be calculated. The magnitude of the ES is classified as large (≥ 0.80), moderate (0.50–0.79) or small (< 0.50) [39].

IBM Statistical Package for the Social Sciences (SPSS) version 26 (IBM Corp., Armonk, NY USA) will be used to perform all statistical analyses. All tests will be one-tailed. A *p*-value < 0.05 will be considered significant for all analyses.

### Economic outcomes

The cost-utility analysis will be conducted in agreement with the intention-to-treat principle. The incremental cost-utility ratio (ICUR) will be calculated by dividing the differences in total costs (i.e. mean costs in the intervention group minus mean costs in the control group) by the differences in QALYs (i.e. mean QALYs in the intervention group minus mean QALYs in the control group). To calculate total costs from a societal perspective, intervention costs, costs of healthcare (i.e. costs of healthcare use and medication), costs for patients and their families (e.g. travelling costs and help received from family and friends), and costs within other sectors (e.g. productivity losses from paid work) will be included. Also total costs from a healthcare perspective will be calculated, which includes intervention costs and the costs of healthcare.

By multiplying resource use by integral cost prices as presented in the Dutch Health Care Insurance Board (CVZ) guidelines on cost studies, costs of healthcare and costs for patients and their families will be calculated [40]. The friction cost method will be used to calculate costs within other sectors [41, 42].

The time horizon will be set at three months follow-up, and therefore neither costs nor effects will be discounted. QALYs will be calculated by multiplying the EQ-5D utility score by the appropriate time period it accounts for. When data are missing on the costs of healthcare, the costs for patients and their families and the costs within other sectors, measured with the iMCQ and iPCQ cost questionnaires, these will be imputed using multiple imputation. This also accounts for missing data on the utilities measured with the EQ-5D.

Non-parametric bootstrapping with 5000 imputations will be used to obtain 95% confidence intervals around the cost and QALY differences. A cost-utility plane will be plotted for the projection of the resulting pairs of cost and effect differences and a cost-effectiveness acceptability curve will be made to reflect the probability of Oncokompas being cost-effective given different willingness-to-pay ceilings [43]. Sensitivity analyses will be conducted focusing on uncertainty in the main cost factors.

## Discussion

This study among patients with incurable cancer will assess the efficacy of the eHealth self-management application Oncokompas on patient activation, general self-efficacy and health-related quality of life, and its cost-utility from a healthcare and societal perspective, compared to care as usual.

Patients with incurable cancer often have unmet needs and prefer to stay in charge of their own life as long as possible. Therefore, it is important that these patients know where to go for advice and guidance. Oncokompas is developed to support patients to adopt an active role in managing their own health and healthcare. By improving patient activation and self-efficacy, Oncokompas could be a solution to meet patients’ palliative care needs. It provides information and advice to empower patients to take better care of themselves and, when necessary, information on where they can find professional help. By improving the provision of support or facilitating patients to find support, eHealth reduces patients’ needs for support [12, 44]. Due to increasing healthcare costs, an essential advantage of eHealth is its cost-saving potential [23]. Oncokompas is based on the stepped care principle, meaning that the application supports patients to undertake actions to control their symptoms, only with professional care if needed. Therefore, it is hypothesized that Oncokompas will improve QALYs at acceptable costs compared to care as usual.

Oncokompas could stimulate patients to discuss symptoms or questions with their healthcare professional that otherwise would remain unmentioned. Previous studies showed that for instance sexuality issues or concerns about the end-of-life are difficult to address for both patients and their healthcare professionals [45–48]. In addition, consultation time is often short, which hampers addressing all relevant issues that a patient might want to discuss [26]. Oncokompas could also help patients to discuss their symptoms with their healthcare professional in a more structured way (e.g. because they might be more aware of their symptoms and also have the possibility to print their results and take this print to their healthcare professional). Another advantage for patients is that they can use Oncokompas at their own home in their own time.

Since Oncokompas includes topics about decisions at the end-of-life, the application could stimulate patients to think about their wishes regarding the end-of-life (e.g. treatment goals or their preferred place of death) and to talk about this to their family, friends, and healthcare professionals. Therefore, Oncokompas has the potential to contribute to the process of advance care planning (ACP). ACP is the process of discussing patients’ preferences concerning their healthcare, so that they receive the end-of-life care they desire [49]. Research showed positive effects of ACP on the quality of care at the end-of-life [49, 50]. ACP could also have a positive effect on the continuity of care (i.e. the information exchange between healthcare professionals to realize optimal integrated care) during the end-of-life. In its turn this is associated with higher quality of care and lower healthcare costs [51, 52].

In three previous studies on Oncokompas among patients diagnosed with glioma, breast cancer, and head and neck cancer, patients reported that they expected that Oncokompas would stimulate them in taking control and acting upon their symptoms [26, 53, 54]. In addition, one of these studies showed that breast cancer survivors’ activation level was significantly higher after using Oncokompas than before [54]. In 2016 a large RCT started to determine the efficacy of Oncokompas on patient activation and cost-utility among cancer survivors [55]; this study is still ongoing.

To summarize, there is a growing interest in eHealth to improve self-management among patients with chronic disease to emphasize the central role of patients in the management of their own disease and to reduce healthcare costs. This study could contribute to the evidence about the effectiveness of tailored eHealth interventions supporting self-management used in palliative care. When the results of this study show that Oncokompas is effective for patients with incurable cancer, this means that the application supports self-management among these patients. This might improve sustainable implementation and maintenance of the application in advanced cancer care.

## Trial status

This study is still ongoing. The recruitment of patients for this study started in January 2019 and is expected to be complete in June 2020. No publications containing the results of this study have been published or submitted to any other journal.

## Supplementary information

**Additional file 1.** SPIRIT checklist.

## Data Availability

The datasets analyzed during the current study will be available in the EASY repository of DANS-KNAW after completion of the thesis that will be written reserving the generated data. The (intellectual) property rights with regard to the generated data will reside at the Vrije Universiteit Amsterdam. Interested parties can request a non-exclusive license for research and educational purposes. The non-exclusive license may be requested only after the completion of the thesis that will be written reserving the generated data.

## Abbreviations

ACP: Advance Care Planning
CVZ: Dutch Health Care Insurance Board
EORTC QLQ C15-PAL: 15-item European Organization for Research and Treatment of Cancer Quality of Life Questionnaire for cancer patients in palliative care
EORTC QLQ C30: 30-item core European Organization for Research and Treatment of Cancer Quality of Life Questionnaire
EQ-5D: 5-dimension EuroQol questionnaire
ES: Effect Size
GSE: General Self-Efficacy
HRQOL: Health-related quality of life
ICUR: Incremental cost-utility ratio
iMCQ: iMTA Medical Consumption Questionnaire
iMTA: Institute for Medical Technology Assessment
iPCQ: iMTA Productivity Cost Questionnaire
LMM: Linear Mixed Models
PAM: Patient Activation Measure
PROMs: Patient-Reported Outcome Measures
QALY: Quality-Adjusted Life Year
RCT: Randomized Controlled Trial
SPIRIT: Standard Protocol Items: Recommendations for Interventional Trials
